# Quality of Life and Weight Loss in Bariatric Patients

**DOI:** 10.1007/s11695-017-2587-8

**Published:** 2017-02-18

**Authors:** Michał Robert Janik

**Affiliations:** Department of General, Oncologic, Metabolic and Thoracic Surgery, Military Institute of Medicine, Szaserów 128 Street, 04-141 Warsaw, Poland

Dr. Scott Shikora

Editor in Chief

*Obesity Surgery*

Dear Dr. Scott Shikora

We are glad that our article has attracted the interest of readers and opened an important discussion about quality of life in bariatric patients. Moreover, we are honoured that our study has raised the interest of Álvares, R. S. R et al. who present their results on the relationship between weight loss and quality of life in group of patients after Roux-en Y Gastric Bypass (RYGB) in long-term follow-up [1]. This cross-sectional study investigated 101 patients who had undergone RYGB from 1998 to 2011 in Brazilian bariatric center. Quality of life (QoL) was assessed by the Moorehead-Ardelt (MA) quality of life questionnaire. In order to investigate the determinants of long-term QoL after BS, they performed a binary logistic regression model with QoL (improved or not improved) as the outcome variable adjusted for age, gender, partnered status %EBMIL, hypertension, diabetes, history of gastroesophageal reflux, smoking status, and physical activity. The results showed that %EBMIL was the only significant quality of life determinant. This observation was discussed with the results presented in our paper titled: “Quality of Life and Bariatric Surgery: Cross Sectional Study and Analysis of Factors Influencing Outcome”. In our study, we performed correlation analysis between total score in MA and the following psychometric variables: %TWL (*r* = −0.122, *p* = 0.36), ΔBMI (*r* = −0.107, *p* = 0.42), and %EWL (*r* = −0.064, *p* = 0.63) [2]. Álvares, R. S. R et al. pointed out that differences regarding the postoperative times of QoL assessment, comorbidities prevalence, and cultural differences explain these different findings. Clearly, those two studies are not comparable. We agree on that. However, few more issues should be discussed.

First, we used correlation analysis. The aim of this statistical method is to quantify the association between two continuous variables. It is not the same method as binary logistic regression which is used to assess the association between dependent categorical variable with one or more predictor (or independent) variables. Please mind that in Álvares, R. S. R et al. study, the dependent categorical variable was defined by authors (improved/non-improved). Different type of statistical method used in the studies can affect the results.

Second, both analyses did not include the information regarding body contouring after bariatric surgery. Many patients after massive weight loss are disappointed due to an excess skin folds. This might have an impact on QoL [3]. It is more likely that patients who are more than 5 years after bariatric procedure underwent also abdominoplasty, especially in country where plastic surgery is very popular [4]. In our opinion, future studies on QoL in bariatric patients should also include information about body contouring after massive weight loss.

Third, both studies were cross-sectional design. This type of study is not intended to assess the impact of weight loss on QoL. The aims in our study were to compare QOL in obese patients 12–18 months after bariatric surgery to control seeking surgery and to investigate which factors influence QOL outcomes in the MA II in obese patients. We found that QoL is significantly better in patients after bariatric surgery (1.70 ± 0.76 in the operated group versus 0.59 ± 1.17 in the control group; *p* < 0.01). We also stated that the fact of underwent bariatric surgery is only significant factor affecting QoL scoring in obese patients (OR 0.113; 95% CI 0.044–0.290). The analysis of correlation between %EWL and QoL scoring was performed in addition. The lack of correlation was consistent with other studies [5, 6]. We decided to present it in the manuscript because we wanted to show that surgeons should not be only a “BMI-hunter”. Yet, there is a need for longitudinal study to obtain a reliable answer on a question about the relationship between weight loss and QoL in bariatric patients.

To sum it up, there is agreement that weight loss leads to better quality of life. Yet, it is not clear what exactly contribute to this improvement. It seems reasonable that the amount of weight loss does not play a significant role after crossing certain threshold. This threshold can be specific for each patient. In our opinion, other factors should be taken into the account like improvement of comorbidities or changes in body image.
